# An assessment of maternal, newborn and child health implementation studies in Nigeria: implications for evidence informed policymaking and practice

**DOI:** 10.15171/hpp.2016.20

**Published:** 2016-08-10

**Authors:** Chigozie Jesse Uneke, Issiaka Sombie, Namoudou Keita, Virgil Lokossou, Ermel Johnson, Pierre Ongolo-Zogo

**Affiliations:** ^1^Knowledge Translation Platform, African Institute for Health Policy & Health Systems Studies, Ebonyi State University, PMB 053 Abakaliki Nigeria; ^2^Organisation Ouest Africaine de la Santé, 175, avenue Ouezzin Coulibaly, 01 BP 153 Bobo-Dioulasso 01, Burkina Faso; ^3^Hospital Central Yaounde, CDBPH Lawrence VERGNE Building 2nd Floor, Avenue Henry Dunant, Messa, Yaoundé, Cameroon

**Keywords:** Maternal, Newborn, Child, Implementation studies, Evidence informed, Policymaking

## Abstract

**Background:** The introduction of implementation science into maternal, newborn and child health (MNCH) research has facilitated better methods to improve uptake of research findings into practices. With increase in implementation research related to MNCH world-wide, stronger scientific evidence are now available and have improved MNCH policies in many countries including Nigeria. The purpose of this study was to review MNCH implementation studies undertaken in Nigeria in order to understand the extent the evidence generated informed better policy.

**Methods:** This study was a systematic review. A MEDLINE Entrez PubMed search was performed in August 2015 and implementation studies that investigated MNCH in Nigeria from 1966 to 2015 in relation to health policy were sought. Search key words included Nigeria, health policy, maternal, newborn, and child health. Only policy relevant studies that were implementation or intervention research which generated evidence to improve MNCH in Nigeria were eligible and were selected.

**Results:** A total of 18 relevant studies that fulfilled the study inclusion criteria were identified out of 471 studies found. These studies generated high quality policy relevance evidence relating to task shifting, breastfeeding practices, maternal nutrition, childhood immunization, kangaroo mother care (KMC), prevention of maternal to child transmission of HIV, etc. These indicated significant improvements in maternal health outcomes in localities and health facilities where the studies were undertaken.

**Conclusion: ** There is a dire need for more implementation research related to MNCH in low income settings because the priority for improved MNCH outcome is not so much the development of new technologies but solving implementation issues, such as how to scale up and evaluate interventions within complex health systems.

## Introduction

With the introduction of implementation science into maternal, newborn and child health (MNCH) research, better methods to improve the uptake, implementation, and translation of research findings into routine and common practices have evolved.^1-4^ Findings from sufficient number of studies have indicated that research evidence can enhance health policy process and development by informing decisions about policy content and direction and evaluating the impact of policy.^5-8^ The World Health Organization (WHO), noted that better use of research evidence in development policy making can save lives through more effective policies that respond to scientific and technological advances.^9^

However, according to Peters and colleagues,^10^ one of the greatest challenges facing the global health community is how to take proven interventions and implement them in the real world. They therefore noted that implementation research, is crucial to meeting that challenge, providing a basis for the context-specific, evidence-informed decision-making needed to make what is possible in theory a reality in practice.^10^ In a recent USAIDS report, it was noted that implementation science is the use of strategies to adopt, adapt, and integrate evidence-based health interventions and policies, changing practice patterns within specific settings.^11^ Implementation research on MNCH therefore, has the potential of generating high quality research evidence that can improve MNCH policy and practice globally. This is why Whitworth and colleagues^2^ argued that the priority for maternal and child survival is not so much the development of new technologies but solving implementation issues. According to them, such implementation research should not only focus the attention of policy makers and implementers, but also improve decision making.^2^

Although implementation research is a relatively new and somewhat neglected field, interest in it is growing, largely in recognition of the contribution it can make to maximizing the beneficial impact of health interventions.^10^ With the increase in implementation research related to MNCH world-wide, stronger scientific evidence are now available and have helped to improved MNCH policies in many countries. Consequently, the total number of maternal deaths worldwide has reportedly dropped by a third within the last decade, although there is yet to be any significant reduction in maternal mortality in most low- and middle-income countries (LMICs).^12^

In Nigeria, with a population of over 160 million and weak health systems, health outcomes especially those related to maternal and child health remains poor. With approximately 2.5% of the world’s population, the country is reportedly having more than 10% of all under-5 and maternal deaths – more than 1 million newborn, infant, and child deaths and more than 50 000 maternal deaths every year.^13-15^ However, there has been some level of reduction in maternal and child mortality with the last few years. The national maternal mortality rate (MMR) reduced from 800/100 000 in 2005^16,17^ to 545/100 000 in 2008^18^ and to 110/100 000 according to the recent Nigeria Demographic and Health Survey (NDHS) 2013.^19^ The 2008 NDHS reported an under five mortality rate (U5MR) of 157 deaths per 1000 live births, suggesting a 22% decline from the NDHS report of 2003 which had shown an U5MR of 201 per 1000 live births.^18,20^ According to the World Bank recent report, the Nigeria U5MR further declined to 117 per 1000 live births in 2013.^21^

Although this reduction in maternal and child mortality in Nigeria could be attributed to implementation of various intervention policies, it is however not very clear as to what extent the policies were informed by research evidence from implementation research. Some implementation research undertaken have attempted to evaluate policy recommendation regarding MNCH to generate more evidence to either sustain or revise existing policy, while others have attempted to implement new recommendations to generate more evidence that will translate into MNCH policies. Till date no attempt has been made to review these implementation studies in order to understand the extent the evidence generated informed better health policy. The objectives of this study are as follows: first, to gain a better understanding of how implementation research has demonstrated the efficacy of available interventions in Nigeria warranting scaling-up, secondly, to review the various MNCH health issue of intervention assessed using implementation research and their implication for policy and practice.

## Materials and Methods

A MEDLINE Entrez PubMed search was performed in August 2015 and studies published in English that investigated maternal and child health in Nigeria in relation to health policy were sought.

We used mainly the PubMed database for extraction of relevant publications principally because in Nigeria and possibly world-wide, studies indexed in PubMed are regarded to have undergone highest form of peer review process and are hence regarded as scientifically reliable evidence. We searched the PubMed for studies from Nigeria undertaken from 1966 to 2015.

The following were the categories and search strategies/key words used and the publications yielded.

Category 1: *Nigeria, health policy, maternal health= *146 publications;

Category 2: *Nigeria, health policy, newborn health= *76 publications;

Category 3: *Nigeria, health policy, child health= *249 publications.

**Figure 1:**
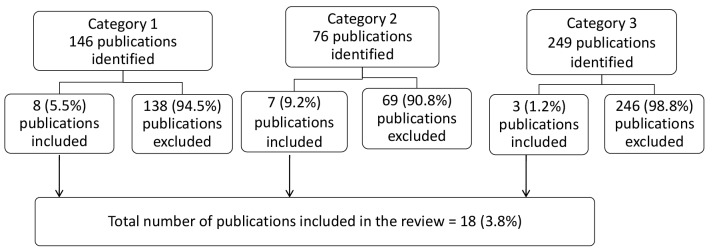
Flowchart of publication identification and selection process.

No software was employed for the search; however the references of all the resulting publications were hand searched for additional studies and information relevant to the review. Publications that did not completely fulfill the study inclusion criteria but adjudged by the authors to contain vital information necessary for narrative aspect of the review were selected and used accordingly. The principal author performed independent data extraction using the predetermined review criteria while two other authors validated extracted data. Authors were satisfied with amount and quality of available information from selected publications and needed not to contact their respective authors/investigators for data confirmation or additional information. All the publications in the three categories were subjected to the following study inclusion criteria:

(*i*) Must be an implementation or intervention research conducted in Nigeria;

(*ii*) Must either have reported the implementation of an intervention to generate evidence to improve MNCH or evaluated an interventions on MNCH already operational in Nigeria;

(*iii*) Must have produced evidence that is relevant to MNCH policy;

Of the 146 publication yielded in category 1, 8 (5.5%)^22-29^ fulfilled the study inclusion criteria regarding maternal health and were selected (Table 1). Out of the 76 publications yielded in category 2, 7(9.2%)^30-36^ satisfied the study inclusion criteria regarding newborn health and were selected (Table 2), while of the 249 publication from category 3, only 3 (1.2%)^37-39^ met the study inclusion criteria regarding child health and were selected (Table 3). The flowchart of the study selection procedure is shown in Figure 1. The selected publications were then grouped according to the following: Author/year of publication; Category of intervention; Health issue of intervention; Evidence-generated; Policy relevant conclusion.

**Table 1:** Profile and characteristics of scientific publications reporting the implementation of an intervention to generate evidence to improve *maternal health* or evaluation of interventions on *maternal health* already operational in Nigeria

**Author/year of publication/reference**	**Category of intervention**	**Health issue of intervention**	**Evidence-generated**	** Implication for policy and practice**
Deller et al 2015^22^	Evaluated MCNH intervention already operational	Task shifting in maternal and newborn health care	Key components that facilitate effective task shifting including policy & regulatory support, education & training, service delivery support & referral systems	Task shifting should be considered as a part of the larger health system that needs to be designed to equitably meet the needs of mothers, newborns, children, and families.
Oseji and Ogu 2014^23^	Evaluated MCNH intervention already operational	Contributions of professional bodies & stakeholders in implementing community-based interventions for MNCH	Reduction of maternal mortality through advocacy, awareness creation, and sensitisation programmes	Health stakeholders impacted positively in the quest for reduction of maternal mortality.
Findley et al 2015^24^	Implemented an intervention to generate evidence to improve MCNH	Integrated MNCH program to improve maternal health outcomes	Integrated MNCH program increased health care utilization	Significant improvements in communities show importance of integrated approach blending supply- and demand-side IMNCH interventions.
Senbanjo et al 2014^25^	Evaluated MCNH intervention already operational	Breastfeeding policy and practices	Breastfeeding practices and policy implementation were suboptimal	There is need for interventions to increase knowledge of the benefits of breastfeeding and to provide support for its longer term duration.
Findley et al 2013^26^	Implemented an intervention to generate evidence to improve MCNH	Integrated MNCH program to improve maternal health outcomes	Infant and child mortality declined greatest in intervention communities.	Participatory and community-based interventions focusing on improved newborn and infant care were effective at improving outcomes in intervention communities
Girard et al 2012^27^	Evaluated MCNH intervention already operational	Maternal nutrition policy and programming	Perceived weak advocacy for nutrition and its role in economic development and the lack of coordination among actors contribute to low prioritization	Advocacy for maternal nutrition could hasten prioritization, coordination, and investment in maternal nutrition
Erim et al 2012^28^	Evaluated MCNH intervention already operational	Use of obstetric care in healthcare facilities	Most of the primary healthcare facilities were unable to provide all basic Emergency Obstetric Care (bEmOC) services	Reducing maternal deaths will require attention to increasing the number of facilities with high-quality EmOC capability
Prata et al 2012^29^	Implemented an intervention to generate evidence to improve MCNH	Community mobilization to reduce postpartum hemorrhage in home births	High level community participation in health care interventions reduce postpartum hemorrhage in home births	Community mobilization can have a significant impact on the successful distribution and uptake of a potentially life-saving health intervention

**Table 2:** Profile and characteristics of scientific publications reporting the implementation of an intervention to generate evidence to improve newborn *health* or evaluation of interventions on newborn *health* already operational in Nigeria

**Author/year of publication/reference**	**Category of intervention**	**Health issue of intervention**	**Evidence-generated**	** Implication for policy and practice**
Ado et al 2014^30^	Evaluated MCNH intervention already operational	National emergency action plan to eradicate polio	Significant improvements in the management of polio eradication initiative (PEI) activities with marked improvement in the quality of supplemental immunization activities (SIAs), as measured by lot quality assurance sampling (LQAS).	Sustained improvement in SIA quality, surveillance, and outbreak response and special strategies in security-compromised areas are needed to interrupt wild poliovirus (WPV) transmission
Ogbolu et al 2013^31^	Evaluated MCNH intervention already operational	Prevention of Maternal to Child Transmission of HIV	Key PMTCT practices are not being adequately translated from research into practice	Strategies derived from an implementation science perspective are suggested as a means to improve the translation of PMTCT research into practice
Adegboye et al 2014^32^	Evaluated MCNH intervention already operational	Childhood immunization uptake and coverage	Statistical significance of the community-survey year interaction term suggests an increase in the odds of a child being immunized over the years and spread over communities	Evidence-based policy should lay more emphasis on mother- and community-level risk factors in order to increase immunization coverage
Okonofua et al 2011^33^	Evaluated MCNH intervention already operational	Advocacy program implementing a policy of free maternal and child health (MCH) services	Advocacy consisted of public presentation on MCH to high-level policymakers, dissemination of situational analysis report, and media publicity	Advocacy and public health education is effective in increasing the commitment of policymakers to provide resources for implementing evidence-based maternal and child health services
Okafor et al 2009^34^	Implemented an intervention to generate evidence to improve MCNH	Risk factors for perinatal mortality associated with anaesthesia for caesarean delivery in patients with pre-eclampsia/eclampsia	Pre-eclampsia/eclampsia continues to be a cause of foetal loss even where essential obstetric services are available.	Early onset management of severe pre-eclampsia with maintenance of adequate placental perfusion during anaesthesia may result in lower perinatal deaths.
Nwogu et al 2008^35^	Evaluated MCNH intervention already operational	Outcome of the Expanded Program on Immunization (EPI)	Results showed that the EPI program had little effect on under-five mortality rate (UFMR)	Nigeria needs a unified approach to healthcare delivery, rather than fragmented programs, to overcome cultural and political divisions in society
Ibe et al 2004^36^	Implemented an intervention to generate evidence to improve MCNH	Comparison of kangaroo mother care (KMC) and conventional incubator care (CC) for thermal regulation of infants <2000 g	Mothers felt that KMC was safe, and preferred the method to CC because it did not separate them from their infants	Where equipment for thermal regulation is lacking or unreliable, KMC is a preferable method for managing stable low birthweight infants.

**Table 3:** Profile and characteristics of scientific publications reporting the implementation of an intervention to generate evidence to improve child *health* or evaluation of interventions on child *health* already operational in Nigeria

**Author/year of publication/reference**	**Category of intervention**	**Health issue of intervention**	**Evidence-generated**	** Implication for policy and practice**
Rowe et al 2012^37^	Evaluated MCNH intervention already operational	Health worker performance after implementing the Integrated Management of Childhood Illness strategy	Performance trends were essentially flat for nearly all outcomes and absolute levels of performance revealed substantial performance gaps.	No evidence that performance declined over 3 years after IMCI training. However, important performance gaps found immediately after IMCI training persisted and should be addressed.
Mafe et al 2005^38^	Implemented an intervention to generate evidence to improve MCNH	Mass delivery of praziquantel among school-aged children in rural communities	Community channel of delivery achieved the best coverage compared to the health facility and school channels	Community channel of praziquantel delivery ensures good coverage of both in and out-of-school children.
Omotade et al 2000^39^	Evaluated MCNH intervention already operational	Treatment regimens for acute diarrhoea in children	Not all types of diarrhoea were recognized as illnesses, and only those considered to be illnesses were treated	The need to adapt health policy and programmes to cultural norms should be addressed to improve the impact of programmes

## Results

Regarding maternal health, five identified scientific publications^22-25,27,28^ reported the evaluation of interventions on maternal health already operational in Nigeria while three^24,26,29^ reported the implemented an intervention to generate evidence to improve maternal health (Table 1). The evaluated MCNH intervention already operational included task shifting in maternal and newborn health care; contributions of professional bodies and stakeholders in implementing community-based interventions. Others included breast feeding policy and practices; maternal nutrition policy and programming and use of obstetric care in healthcare facilities. The implemented intervention to generate evidence to improve maternal health outcomes included integrated MNCH, and community mobilization to reduce postpartum hemorrhage in home births (Table 1). These studies generated high quality policy relevance evidence which indicated significant improvements in maternal health outcomes where the studies were undertaken.

Table 2 presents five identified scientific publications^30-33,35^ reporting the evaluation of interventions on newborn health already operational in Nigeria. There were also two publications^34,36^ that implemented an intervention to generate evidence to improve newborn health. The existing interventions evaluated included: national emergency action plan to eradicate polio and prevention of maternal to child transmission of HIV. Others included childhood immunization uptake/coverage; and free maternal/child health services. The implemented intervention to generate evidence to improve newborn health outcomes included, early onset management of severe preeclampsia with maintenance of adequate placental perfusion; and comparison of kangaroo mother care (KMC) and conventional incubator care (CC) for thermal regulation of infants < 2000 g (Table 2). The results of these studies indicated that the interventions gave rise to significant improvements in newborn health outcomes.

Of the three scientific publications reporting interventions to improve child health in Nigeria, two^37,39^ evaluated intervention programmes already operational while one^38^ implemented an intervention to generate policy relevant evidence (Table 3). The existing interventions evaluated included health worker performance after implementing the integrated management of childhood illness strategy and treatment regimens for acute diarrhea in children. The implemented intervention to generate evidence to improve child health outcome was on mass delivery of praziquantel among school-aged children. These studies generated high quality policy relevance evidence which indicated significant improvements in child health outcomes (Table 3).

## Discussion

This review was designed to provide more insight into the process of evidence-informed policymaking and knowledge transfer/exchange based on implementation research regarding MNCH in Nigeria. The findings of this review showed that implementation research still remains one of the most effective processes of demonstrating the efficacy of available or new health interventions in order to improve access to, and the use of, these interventions to improve MNCH. All the studies reviewed reported tremendous improvements in maternal and child health outcomes as a result of the interventions implemented. There is therefore the need for scaling up of the intervention at country level. In recent times there is a world-wide consensus regarding the dire need for scaling up life-saving interventions as a way to reduce maternal and infant mortality and morbidity.^2,4^ Implementation research is therefore gaining global attention due to the large underuse of several life-saving interventions in LMICs.^10^ Because scaling up proven MNCH interventions are inevitable, this review makes a case for more implementation studies that will identify and address implementation bottle-necks and the major barriers that hamper access to interventions.

Out of the combined total of 471 publications found from the MEDLINE search related to MNCH and policy in Nigeria, only 18 (3.8%) fulfilled the inclusion criteria and found to be implementation research with policy relevant outcomes. The implication of this finding suggests that inadequate number of implementation research relevant to MNCH policy has been undertaken in Nigeria.

According to Averting Maternal Death and Disability Program (AMDD),^40^ one reason for insufficient MNCH implementation research in LMICs is because it is still relatively new to the field of public health. Even though it offers multiple approaches to building the more detailed and specific evidence base needed to answer critical questions about how to promote equitable access to maternal healthcare.^40^ Peters and colleagues^10^ added that implementation research, continues to be a neglected field of study, partly because of a lack of understanding regarding what it is. They also noted that it is due to a lack of investment in it.^10^

In Nigeria, there is grossly inadequate funding support for implementation research and this appears to be a common scenario in most LMICs. In a recent report, Dean and colleague^41^ noted that although a substantial proportion of maternal and child deaths in LMICs are preventable, progress in reducing these deaths is far too slow due to the bias that remains in health care and research investment. Dean and colleagues^41^ further cited an instance where 7.6 million children died worldwide in 2010 which is equivalent to global deaths due to cancer and slightly higher than deaths due to heart disease.^42,43^ Yet funding favors breakthrough research for cancer and heart disease, while implementation research and delivery for maternal/child health is sidelined.^42,43^

It is of interest to note from this review that of the 18 selected studies, 12 (66.7%) were published between years 2012-2015. The finding suggests that there is increasing interest in implementation research related to MNCH in Nigeria within the last few years. This is consistent with what is obtainable in parts of Africa and other LMICs as attested by some recent studies which proved that there is increasing recognition of the importance and value of implementation research to improve MNCH policy and practice.^4,44-47^ According to WHO,^48^ implementation research is increasingly being recognized as one of the most important interfaces between the availability of tools, strategies and interventions and their use within health systems and control programmes. This is because limited uptake of research findings and innovations in real-world settings has led to mounting interest in implementation research for public health.^48^ Furthermore, in a very recent study on critical maternal health knowledge gaps in LMICs, Kendall and Langer^49^ noted that the global maternal health researchers consulted placed high priority on implementation research to improve the delivery of existing evidence-based maternal health interventions. This is consistent with the results of a recently published international survey on priorities for maternal and perinatal health research.^50^

The studies reviewed in this report evaluated more that 14 MNCH policy issues of high priority in Nigeria including: task shifting, community-based interventions, breastfeeding policy and practices, maternal nutrition policy, use of obstetric care in healthcare facilities, community mobilization to reduce postpartum hemorrhage in home births, emergency action plan to eradicate polio, prevention of maternal to child transmission of HIV, childhood immunization uptake and coverage, free maternal and child health services, management of severe preeclampsia, KMC, integrated management of childhood illness strategy, and treatment regimens for acute diarrhea in children. One very interesting feature of all the studies is that they all generated high quality policy relevance evidence which indicated significant improvements in MNCH outcomes in localities and health facilities where the studies were undertaken.

These findings further provide a confirmation of the value and necessity of implementation or intervention studies in strengthening the provision of research evidence that will not only produce strong policies but effective healthcare practice. The findings from these studies provided evidence which informed three important policy documents in Nigeria including *the Nigeria’s Call to Action to Save Newborn Lives*,^51^ *the Saving One Million Lives Initiative of Nigeria*^52^ and *The National Health Bill, 2014*.^53^ This is because the findings of the studies were part of the evidence incorporated in the recommendations in these three important documents.

It is therefore pertinent to state that more implementation research in MNCH is needed not only in Nigeria but in other LMICs where health systems are weak and where maternal and child health outcomes are poor. The Centre for Population and Environmental Development,^54^ noted in their policy brief on the improvement of MNCH in Nigeria that less attention has been paid to implementation research in Nigeria. This entails the production of evidence on the best ways to support the adoption of, and optimize use of innovations in MNCH care. Therefore the ability to test diverse MNCH implementation pathways and to identify what works in rural community settings is critical to the improvement of MNCH care. According to WHO,^48^ implementation research will provide evidence on the best ways to support the adoption of, and optimize use of innovations and holds promise for scale-up and for greater commitment and investment.

## Study limitations

This study had two main limitations. First, we used only the PubMed for data extraction. Although PubMed is regarded as one of the most outstanding and globally recognized easily assessable databases for health sciences publications. Our inability to search other databases may have resulted in missing additional relevant publications. We advocate the inclusion of other databases in future studies. Another limitation to this study has to do with the scope of the reviewed publications. Every study reviewed was conducted only in as section of Nigeria. Consequently it may be inappropriate to generalize the findings because of the diverse socio-economic and cultural settings of Nigeria. There may be a need to repeat some of the studies in other parts of the country to see if there will be similar or contrary outcomes.

## Conclusion

There is no doubt that there is a dire need for more implementation research related to MNCH in LMICs including Nigeria. This is because the priority for maternal and child survival is not so much the development of new technologies but solving implementation issues, such as how to scale up and evaluate interventions within complex health systems.^2^ Not only do we need to identify the most effective ways to deliver, scale up and sustain both basic and comprehensive emergency obstetric care, especially for postpartum hemorrhage and preeclampsia, but implementation research is needed to ensure we deliver the right packages of care at the right levels of care.^3^

However, it must be noted that generating the necessary robust evidence for improved MNCH outcome is not easy. The reason is because implementation research is not free from limitations and challenges. It will certainly present the same problems as other types of research. Most obviously, findings of implementation research need to be taken up by the implementers to close the gap between evidence generated by the implementation researchers and practices.^55^ Furthermore, evaluation and implementation research of the delivery of MNCH interventions that focus on impact, specifically those assessing changes in morbidity or mortality, is advocated since they have been deemed critical to determine the effectiveness of programs being implemented.^56^ Finally, in the Federal Ministry of Health of Nigeria publication on saving newborn lives,^57^ stakeholders are encouraged to conduct locally driven implementation research and act on the results as well as prioritize use of local data for decision making and implementation research to fill knowledge gaps for maternal, newborn, and child health.

## Acknowledgements

This study was one of the outcomes of the “*Moving Maternal, Neonatal and Child Health Evidence into Policy in West Africa”* ​​(MEP) project undertaken by West African Health organization (WAHO) funded by International Development Research Centre (IDRC) Canada (Reference: IDRC 107892_001).

## Ethical approval

This review was a key component of the** “***Moving Maternal, Neonatal and Child Health Evidence into Policy in West Africa”* ​​(MEP) project undertaken by West African Health Organization (WAHO). Ethical clearance was obtained from the University Research Ethics Committee of Ebonyi State University Nigeria (the institution of the principal author).

## Competing interests

The authors declare no competing interest.

## Authors’ contributions

All authors participated in the design and development of the study. CJU, IS and PO reviewed the studies selected and agreed on the studies included. CJU drafted the manuscript, with contributions from IS, PO and NK. All authors made inputs to the final manuscript.
